# Evaluation of a training programme for Pharmacist Independent Prescribers in a care home medicine management intervention

**DOI:** 10.1186/s12909-022-03575-5

**Published:** 2022-07-15

**Authors:** L. Birt, L. Dalgarno, C. M. Bond, R. Holland, D. P. Alldred, C. Hughes, A. Blyth, L. Watts, D. J. Wright, on behalf of the CHIPPS team

**Affiliations:** 1 School of Health Sciences, Faculty of Medicine and Health Sciences, University of East Anglia, Norwich Research Park, Norwich, NR4 7TJ UK; 2 School of Allied Health Professions, University of Leicester, Leicester, UK; 3 School of Medicine, Medical Sciences and Nutrition, University of Aberdeen, Aberdeen, UK; 4 Leicester Medical School, University of Leicester, Leicester, UK; 5 School of Healthcare, University of Leeds, Leeds, UK; 6 School of Pharmacy, Queen’s University Belfast, Belfast, UK; 7 School of Pharmacy, University of East Anglia, Norwich Research Park, Norwich, UK

**Keywords:** Pharmacist, Training, Independent prescribing, Care homes, Deprescribing, Professional competency, Medicine management

## Abstract

**Background:**

The provision of independent prescribing rights for United Kingdom (UK) pharmacists has enabled them to prescribe within their area of competence. The aim of this study was to evaluate an evidence-based training programme designed to prepare Pharmacist Independent Prescribers (PIPs) to safely and effectively assume responsibility for pharmaceutical care of older people in care homes in the UK, within a randomised controlled trial.

**Methods:**

The training and competency assessment process included two training days, professional development planning against a bespoke competency framework, mentor support, and a viva with an independent General Practitioner (GP). Data on the PIPs’ perceptions of the training were collected through evaluation forms immediately after the training days and through online questionnaires and interviews after delivery of the 6-month intervention. Using a mixed method approach each data set was analysed separately then triangulated providing a detailed evaluation of the process. Kaufman’s Model of Learning Evaluation guided interpretations.

**Results:**

All 25 PIPs who received the training completed an evaluation form (*N* = 25). Post-intervention questionnaires were completed by 16 PIPs and 14 PIPs took part in interviews. PIPs reported the training days and mentorship enabled them to develop a personalised portfolio of competence in preparation for discussion during a viva with an independent GP. Contact with the mentor reduced as PIPs gained confidence in their role. PIPs applied their new learning throughout the delivery of the intervention leading to perceived improvements in residents’ quality of life and medicines management. A few PIPs reported that developing a portfolio of competence was time intensive, and that further training on leadership skills would have been beneficial.

**Conclusions:**

The bespoke training programme was fit for purpose. Mentorship and competency assessment were resource intensive but appropriate. An additional benefit was that many PIPs reported professional growth beyond the requirement of the study.

**Trial registration:**

The definitive RCT was registered with the ISRCTN registry (registration number ISRCTN 17,847,169).

**Supplementary Information:**

The online version contains supplementary material available at 10.1186/s12909-022-03575-5.

## Background

Care homes for older people provide support for those individuals who require 24-h care either with or without nursing. Although dementia is the most common reason for admission [1], many residents have multiple morbidities and associated medicines. The average number of medicines prescribed regularly to residents’ ranges from 6 to 9 [2, 3]. Research in the UK identified 70% of residents were exposed to a medicines-related error daily resulting in a government call for interventions to reduce this [2].

The most common intervention to optimise medicines use in care homes has been the deployment of pharmacists who draw on their clinical skills of patient-centred history taking, diagnostic reasoning and pharmacological knowledge to review medication and provide advice on medicines systems [4]. Whilst shown to reduce the number of prescribed medicines and improve medication appropriateness, current models have failed to adequately demonstrate improvements in clinical outcomes. Identification of more effective models of care have therefore been recommended [4].

### Study and training context

The legal provision of prescribing rights to pharmacists in the UK [5] allows them to prescribe and make changes to prescriptions without authorisation from a medical practitioner and therefore to assume responsibility for medicines optimisation activities within care homes. The UK National Institute for Health Research (NIHR) funded the Care Homes Independent Pharmacist Prescriber Study (CHIPPS) to determine the effectiveness and cost-effectiveness of this new model of care [6, 7]. The CHIPPS intervention involved a triad of stakeholders: a Pharmacist Independent Prescriber (PIP), General Practitioner (GP) and staff in the care home from which residents were recruited. All PIPs had a postgraduate qualification in Independent Prescribing and took responsibility for medicines management for participating residents and offered general support and training to the care homes. Early-stage stakeholder engagement identified a need for reassurance regarding pharmacist independent prescribers’ competence to undertake this role in such a frail and elderly population [8]. The legislation enabling independent prescribing by non-medical prescribers states that prescribers only prescribe within their area of competence [9]. Consequently, it is usual to undertake training prior to undertaking the role, this was the aim of the CHIPPS pharmacist training.

A literature review identified limited evidence about the training needs for pharmacists working within care homes, possibly because without prescribing rights, recommendations are checked and authorised [10]. However, in the CHIPPS study pharmacists were autonomous practitioners, therefore requiring assessment of competence before commencing the role.

Extensive multidisciplinary team engagement followed by feasibility testing with four pharmacist prescribers resulted in a training package for use within the main trial [11]. Training was delivered by a multidisciplinary team from across primary care, including senior experienced care home pharmacists, general practitioners and consultant geriatricians. Each PIP undertook two days’ face-to-face training primarily to cover the management of prescribing and deprescribing (stopping problematic medicines) for older people with complex health needs. Teaching methods included using exemplar real life case studies, personal reflection and discussion on managing challenging prescribing activity. In person training was followed by four days to develop relationships with the care homes, medical practices and community pharmacists with whom they would be working (see supplementary file 2 CHIPPS competency framework and training programme). The PIP, or their employer, was reimbursed for this time. PIPs were each allocated a mentor, an experienced pharmacist, who worked with them using the competency framework to create a professional development plan. PIPs subsequently collated a portfolio of evidence to demonstrate competence [11]. They then undertook a viva with an independent GP, to sign off their competence in independent prescribing in care homes.

Within the CHIPPS study PIPs delivered the intervention over six-months in line with an agreed service specification, including agreeing a care plan with residents, care staff and the GP and ensuring regular monitoring of medicines (see supplementary file 1 CHIPPS Intervention Service Specification). The feasibility study [12] found that PIPs wanted more instruction on how to use pharmaceutical care plans (PCPs: a written individualised medicine plan with clear therapeutic goals) so this was added to the training (see supplementary file 2 CHIPPS competency framework and training programme). The 2-day face-to-face training also included sessions specific to the research aspects of the CHIPPS intervention.

This paper reports an evaluation of the evidence-based training programme designed to prepare Pharmacist Independent Prescribers (PIPs) to safely and effectively assume responsibility for pharmaceutical care of older people in care homes in the UK, within a randomised controlled trial.

## Method

### Design

Data were collected as part of the trial process evaluation [13]. English ethical approval was gained from East of England Cambridge Central Research Ethics Committee 17/EE/0360 (28.11.2017); this applied to research undertaken in Northern Ireland. The Scottish ethical approval was gained from Scotland A research Ethics Committee 17/SS/0118 (07.12.2017).

After an inductive analysis we deductively applied Kaufman’s Model of Learning Evaluation [14] which, through five levels of consideration, evaluates training from the learner’s perspective. This enabled us to separate training input from training process, and micro-outcomes (specific to PIPs) from macro-outcomes (relating to GPs, care home staff and residents, and any societal benefits). Kaufman’s Model was appropriate to understanding the PIPs’ views on resources used in training and how training was delivered; and whether there was new learning, whether such new learning was used when delivering the CHIPPS service and the outcomes from new learning for the PIP and other stakeholders.

### Recruitment and sample

PIPs were recruited to the CHIPPS study [7] with the 25 allocated to the intervention arm receiving training for their role. They attended the in-person 2-day training event and had additional paid time for developing personal competency. They are the sample for this evaluation. To triangulate the PIPs accounts we also draw on data from questionnaires and interviews completed by GPs and care homes staff as part of the study process evaluation [13].

### Data collection and analysis

#### Post-training day evaluation questionnaires

At the end of the second training day, PIPs were invited to complete an evaluation questionnaire. This included items on relevance of each topic delivered, as well as efficacy and timing of delivery. Items were rated using a 5-point Likert scale to rate aspects from ‘strongly disagree’ to ‘strongly agree’. These data were collated and tabulated using Excel. Descriptive statistical analysis undertaken. Open text responses were analysed alongside interview data (see below).

#### Post-intervention questionnaire

After delivering the six-month intervention PIPs, GPs and care home managers were invited by email to complete an online questionnaire on their overall experiences [13]. The questionnaire drew on items from the NoMAD questionnaire [15] designed to examine normalisation of new knowledge and skills in the workplace questionnaires contained items on the activities undertaken by the PIP, impact of intervention on relationships, and usefulness of the intervention. GPs were asked if the PIP appeared sufficiently trained for the role. The PIP version also included items relating to perception of training (: sufficiency and usefulness of the training event; usefulness of mentoring prior to sign-off of competence; and usefulness of mentoring during delivery of the intervention. Likert scale responses were collected and tabulated in Excel. Descriptive statistical analysis was undertaken. Open text responses were analysed alongside interview data (see below).

#### Post-intervention interviews

At the end of the 6-month intervention PIPs, GPs and care home staff in the intervention arm of the study were invited by email to take part in a semi-structured interview, either in person or by telephone. The PIP interview topic guide included eight questions related to training and mentorship (supplementary file 3 CHIPPS interview topic guide). These probed PIPs views on relevance and usefulness of the training and assessment of competency process in relation to their prior knowledge and when delivering the intervention. The GP and care home staff topic guide contained questions about relationships with the PIP and satisfaction with the PIP service. Following consent, interviews were audio recorded and data were transcribed and managed in NVivo [15]. Data were inductively, thematically analysed and then PIP data were deductively explored using Kaufman’s Model of Learning Evaluation [14] to contextualise PIPs experiences within evaluation domains. Table 1 summarises the links between our evaluation and Kaufman’s Model.

**Table 1:** Linkage between CHIPPS training evaluation data and Kaufman’s Model of Learning Evaluation

Kaufman’s Model of Learning Evaluation	CHIPPS training evaluation analysis questions	PIP data
Level 1a Input	Did we deliver topics which were useful?	Pre-training preparation *‘Preparing a Case Study that I brought to that day was good just to sort of reframe how you think about things and I suppose feeling confident to make the actions really’* Interview PIP17 *‘I felt the input from geriatricians of significant benefit. In particular discussing case studies and down titrations of various medications in real life practice.*’ PIP15 *‘I found the sessions considering treatment of Dementia in Parkinson's and also the psychiatrist’s session on reviewing and reducing antipsychotics very useful. I didn't have this knowledge’* PIP 22 Areas to improve *‘In Scotland some of the Mental Health legislation differs from how you do things in England so I recall there was a lot of talking about incapacity and certifications and documentation was slightly different …just maybe more inclusive in terms of the documentation and things’* PIP15 *‘Maybe a bit of negotiation training’* PIP22
Level 1 b Process	Did we deliver training in effective ways?	Usefulness of mentor *‘It was actually the mentor chatting around situations she’d encountered, and what we could do, and how we should approach it, that was beneficial, because I wouldn’t have considered how I would go about going in and introducing myself, the mentor giving us these tips on how to build these relationships, has definitely helped’* PIP14 Time to prepare for assessment of competency *‘There was a lot of self-directed work to do and I think this was too much- some more of this could have been covered in the training’* PIP13 Areas to improve *‘Information about the study procedures was helpful but there was an element of duplication’* PIP8
Level 2 Acquisition	Did the PIPs acquire new knowledge?	Refreshing knowledge *‘It certainly refreshed my knowledge, and I’m probably a bit more geared up to do the nursing home reviews’* PIP21 *‘It was good for me to do it because it has made me think about things, but I knew I needed to refresh some things’* PIP2 Increasing competency *‘There were some clinical things that covered areas I wasn’t very competent for example dementia, and the only time I called my mentor was when I had a question about ‘is it okay for me to wean a patient off this particular drug?’ so I obviously learnt from that and got more confident’* PIP8 Areas to improve *‘I wouldn’t say it was a futile exercise [completing professional competency portfolio] it was good to do it, so I won’t really get rid of that but I would definitely make it a lot more concise, a bit more smarter’* PIP16
Level 3 Application	Did the PIPs apply new knowledge?	Making medication changes *‘Quite a lot of medicines were PRN anti-psychotics which is one of the things we done on the training and once the Carers were aware of what they should be looking out for and me making sure that the MAR chart said ‘please follow the PRN protocol more closely’ quite a lot of patients managed to come off them and their sleeping tablets’* PIP19 *‘Covert meds I had been doing already, but it made me reflect, we shouldn’t be leaving them for a year, they need reviewed sooner and do they need the medicine at all, so I think it made me be more thorough as a prescriber and a pharmacist’* PIP22 Increasing confidence *‘There were a few people we’d got off a medication, antipsychotics particularly, because that’s something I probably wouldn’t have touched, but after having the training session, and the group discussions, and more of an awareness, I felt more comfortable* ‘ PIP14 *‘I’d certainly challenge the consultant more, I was a bit more confident, having had the training I’m thinking, hang on a minute, maybe we don’t want to do this, we don’t want to do that’* PIP21
Level 4 Organisational results	Were there impacts on the intervention and stakeholder?	GP reactions *‘PIP knew about nursing home prescribing very well. Offered different options for covert medication which was very helpful. Wrote in the notes clear plans from discussion with staff, patients and family’* GP16 *‘PIP stopped medication that didn’t need to be given; a couple of patients with low blood pressure, picked up that they didn’t need to be on so much medication, that theoretically reduces the risk of falling’*. GP8 Improved review *‘I try and look in advance of 6 monthly review, when did resident last have a blood test, or is there a psychiatric letter, if I see some drugs that I’m thinking, I’m not sure about that one, I’m probably faster at it, I’m thinking right, I’ve got that knowledge in my head, it’s a bit fresher’* PIP21 Benefit to resident *‘There were people that we took off [antipsychotics] and they were actually brighter and happier, and more smiley and that is definitely good’* PIP14
Level 5 Societal/customer consequences	Were there impacts on wider organisations?	Knowledge carried forward *‘I am in a new job now and based on the experience [in CHIPPS] I’ve delivered my own model of working in my new job as a Care Home Pharmacist’* PIP16 PIP sharing expert advice with GPs changed practice *‘I did start to change my practice when I prescribed a cream for a course of treatment I actually put on the directions ‘discard after a week’ or ‘discard after a fortnight’.* GP19 *‘There was a lot of links that they gave us at training days which were very useful, I shared a lot of this at our clinical meeting, areas that are very good to have a reflect on, we’ve had a few different meetings on how we go forward with all the frailty’* PIP22

## Results

Twenty-five PIPs undertook the training days and all completed the post-training evaluation forms. Post-intervention all 25 were invited to take part in a questionnaire and interview. 13 completed both the post-intervention questionnaire and interview; three completed only the questionnaire; and one completed only the interview, meaning data was available from 17 PIPs. Three PIPs stopped delivering the intervention before three months; they did not respond to the recruitment to questionnaire or invitation to interview; another five PIPs did not respond to recruitment emails. There were no notable differences between the demographics of PIPs interviewed and those not interviewed. Table 2 contains details on the characteristics of the PIPs across data collection methods.

**Table 2:** PIP characteristics across data collection methods

	**Post-training evaluation form**	**Post-intervention questionnaire**	**Post-intervention interview**
Number PIPs	25	16	14
Mean time registered as pharmacist	19 years (5–40 years)	21 years (8–40 years)	20 years (10–36 years)
Mean time qualified as prescriber	52 months (4 years 3 months)	65 months (5 years 4 months	58 months (4 years 8 months)
Previous care home experience	11 (44%)	6 (37.5%)	7 (50%)

Eight GPs and 2 care home staff completed the post-intervention questionnaires. Eight GPs and 15 care home staff were interviewed; full characteristics of GP practices and care home are reported in supplementary file 4 Demographic information on triads and participants. Triad number and length of time as an independent prescriber (IP) are used to identify illustrative quotes from PIPs; GP and care home staff are identified by triad number only. Results are presented under the five domains of Kaufman’s model.

### Domain level 1a input—were training resources suitable and appropriate?

**Fig. 1:**
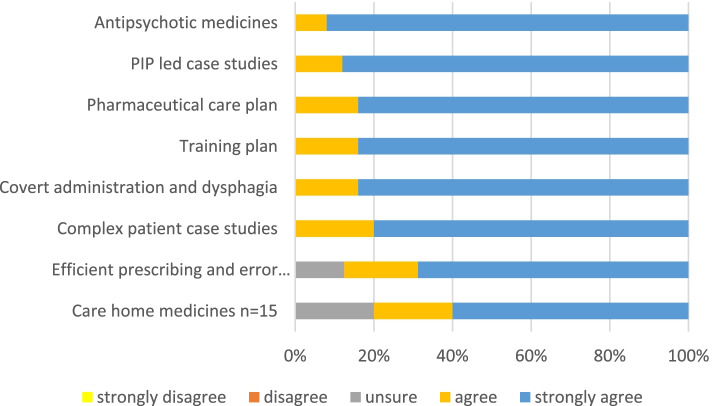
PIP Post-training evaluation on whether a topic should be covered in training (*N* = 25)

The post-training evaluation forms responses (see supplementary file 5 Post-training evaluation responses) on topics included in the clinical sessions indicated strong agreement that all topics should be covered as shown in Fig. 1.

In post-intervention questionnaire item, ‘Was the pre-service delivery training provided sufficient preparation for the role: yes, in-part, no? 13 PIPs replied Yes, 3 replied in-part. In post-intervention questionnaire open text boxes PIPs predominately reported they enjoyed the case studies, finding the training from the geriatric specialists particularly helpful, ‘*I found the peer review of the case studies helpful as it was useful to learn from colleagues and experienced geriatricians’* PIP4 IP1yr.

There were differing opinions on the usefulness of training sessions relating to delivering the research project. In the post-training evaluation form item, ‘Should training cover Research issues & practical procedures*?*’ (Likert 5 point scale) 21 PIPs strongly agreed and 4 agreed. In post-intervention questionnaire open text boxes on item ‘What was the least useful aspect of training?’ Four PIPs referred to study procedures 2 found the talk on completing PCPs least useful, one stated duplication in training on study procedures and one identified that study procedures need to be delivered ‘ *Explaining all the process and ethics can feel the least beneficial part of the training but it has to be completed.*’ PIP15 IP6yrs. Yet in interviews some PIPs suggested that training should include more detail on completing PCPs and include information on communication and relationship-building.

### Domain level 1 b process – was the training well delivered?

In post-training evaluation the majority of PIPs stated the training was delivered effectively and that time allocation was appropriate, see supplementary file 5 post-training evaluation responses. At interview, one PIP suggested that a refresher day part-way through the intervention would have been helpful. Several PIPs had to travel from Northern Ireland and Scotland to attend training sessions held in Norfolk (England) and while all appreciated the face-to-face delivery, this was identified as requiring additional personal resources. For example, although PIPs were paid for attending training, some had to organise domestic situations to be away from home.

In post-intervention questionnaires, PIPs reported that mentor support prior to assessment of competence was useful: 6 very useful, 4 useful, 5 neutral, 1 not very useful. This triangulated with positive comments at interview about the mentorship, the competency framework and assessment of competencies identified as requiring development against the framework: ‘*I quite liked filling it in* [portfolio] *going through it, because it made me realise I knew more than I thought I did, I got quite a bit from it*’ PIP11 IP3yrs.

The viva with the independent GP to sign off competency prior to commencing delivery of the intervention was welcomed by PIPs. In interviews many reported enjoying the dedicated time to talk to another professional about their knowledge and skills. Importantly PIPs acknowledged that this assessment gave them confidence in their capabilities: ‘*I really liked the fact that we were signed off by somebody … it kind of felt a bit more like ‘yeah okay they think you can do this job now*’’ PIP19 IP8yrs.

The need for mentor support decreased as PIPs delivered the intervention with post-intervention questionnaires indicating 4 PIPs identified mentor support during the intervention as very useful, 3 as useful, 6 neutral, 3 as not very useful. However, the expertise of the mentor was acknowledged:

‘*It was useful to have somebody that had a lot of experience in the field …. you don’t know what you don’t know so I had developed what I thought and then it was just further questioning and pointing me in the right direction to add another couple of bits’* PIP17 IP7yrs

### Domain level 2 acquisition – to what extent did PIPs acquire new knowledge and skills?

All PIPs were assessed as competent following their viva and within expected timelines. All identified new areas of learning even those who had long-standing experience as independent prescribers, or working in care homes:

*‘I already have 6-monthly reviews with the care home staff and being a prescriber, I felt confident making a decision about certain drugs, but there were certain ones that I would have held back from, antipsychotics particularly, that’s something I probably wouldn’t have touched… but after having the training session, and the group discussions, and more of an awareness, I felt more comfortable*’ PIP14 IP20yrs

### Domain level 3 application – to what degree did PIPs use their learning in their role on the intervention?

In interviews, several PIPs spoke of new knowledge enabling them to have confidence in prescribing and deprescribing in care homes, ‘*confidence to know that it’s fine to take them [medicines] away*’. A few acknowledged that confidence developed over a few weeks:

*‘I was less confident at the beginning than later on, I suppose that was almost the sort of development stage and by the time we got into the second three months you are obviously still learning as you go along but I was more sort of in the swing of it’* PIP8 IP1.5yrs*.*

### Domain level 4 organisational results – did the PIP training impact on the implementation of the intervention and triad stakeholders?

In the post-intervention questionnaire, all 8 GPs agreed the PIP appeared sufficiently trained for the role. At interview, GPs reported confidence in the PIPs knowledge ‘*Very knowledgeable… pitched her advice giving just right—informed me directly when there was a safety issue but on other issues emphasized options that could be taken’ GP6.*

GPs and care home staff reported at interview that they perceived an increase in medication safety due to the PIP activity. Deprescribing could improve resident quality of life, ‘*reviewing the medication, and seeing do they really need to be on this medication… there was a couple that went on the Accrete (*for vitamin D and calcium deficiency*) for their high risk of falls, that was good as well, that helps me for my falls analysis, that’s another improvement made*’ Care home14.

### Domain level 5 societal consequences – How did the PIP training impact on society at large?

In some triads, the PIPs retained increased responsibility after the intervention ended. One reported that they had developed confidence and in-depth knowledge which enabled them to challenge a prescription from a hospital consultant as they knew the dose was incorrect for that resident. A wider consequence was that some PIPs retained the role of being the GP practice-care home link, facilitating the reduction of GP workload post-intervention.

## Discussion

This evaluation of the bespoke training provided for pharmacist independent prescribers was sufficient to enable them to take responsibility for care home residents’ medicine review and medication management. The professional learning and confidence they developed had impact beyond the CHIPPS intervention. The use of a tailored personal development programme acknowledged pharmacists’ individual prior skills and knowledge. A novel aspect of this research-based training programme was that pharmacists underwent a viva with an independent GP experienced in older people medicine management before delivering the research intervention. This facilitated the implementation of the CHIPPS intervention as GPs and care home staff had confidence in the pharmacist’s clinical competence. This method of training and assessment of competency has transferability to other intervention studies where health professionals are delivering medical interventions in unfamiliar health contexts.

The training programme was developed to equip pharmacists with the professional competence to undertake medicines management for older people living in care homes. Formal training on administration of specific medicines is not unusual, but our training had a focus on the more complex aspects of prescribing and deprescribing in older people with multimorbidity, a relatively new role for pharmacists [16] and one which could challenge another professional’s decision-making. The study process evaluation found there was extensive medication-related activity from all PIPs across all therapeutic groups suggesting that new learning was applied. A vital document in the study was the PCP completed by the pharmacist for each resident. The PCP was relevant to professional accountability and audit but was also a key research study document enabling researchers to analyse prescribing and deprescribing activity. Our results highlighted that PIPs perceived they needed training on the use of the PCP. This resonates with work by Benson et al. who used a Delphi study to reach consensus on the training needs of primary care pharmacists [17]. In that study formulating, implementing and documenting care plans was identified as a training need.

Another intended outcome from the training programme was to educate the pharmacists in the research process and their role within it. While pharmacists are likely to be interested in and supportive of research, it is less likely that they have personal experience of research procedures [18]. In our study immediately after the training days pharmacists agreed that information on research processes was required. However, during post-intervention data collection, the PIPs’ reflections on the training now focused on the benefit they had gained from the elements relating to prescribing and deprescribing for older people and the time to undertake personally directed professional development. It is likely that the research processes had become embedded in everyday practices after six-months of delivering the intervention, but the more tangible transferable generic skills of medicines management and deprescribing remained important to these pharmacists as health care professionals. This reflects suggestions by Sargeant et al. that professional development should be grounded in everyday practice and be of direct benefit to patients if it is also to support quality improvement [19]. Clearly, were this intervention to be rolled out, the research elements of the training programme would be unnecessary.

The interprofessional elements of the training programme, with sessions led by hospital geriatricians and GPs, and a competency assessment by a GP were aspects particularly welcomed by the PIPs and identified as supporting clinical skill development. This supports other calls for training programmes to integrate interprofessional teams and practices [20, 21].

PIPs suggested areas where the training could be improved; these focused predominantly on accessibility to training sessions and more detail on how to build strong relationships, especially where the PIP had not worked previously with the GP. While remote training is becoming the norm and would remove travel demands, there remain challenges using remote delivery methods [22, 23]. However, a refresher mid-way through the trial might be successfully managed remotely as group cohesion would have been developed.

### Strengths and limitations

A strength of the study is that post-intervention data collection was undertaken soon after the pharmacist ended the intervention thereby reducing likelihood of recall bias. However, this means there are no data on whether the benefits reported are enduring. Further work would be required to evaluate long term impact. A limitation is that a full data set was not collected from all PIPs who delivered the intervention, however, the sample was representative of PIP characteristics including length of experience as a pharmacist and an independent prescriber.

## Conclusions

This interdisciplinary, empirically based training programme successfully enabled pharmacist independent prescribers to further develop clinical decision-making skills which enabled them to have the confidence and skills necessary to take responsibility for prescribing and medicines management in care homes. The focus on complex deprescribing was an area new to several pharmacists but the provision of a competency framework and assessment meant they all expressed confidence in delivering this intervention. Furthermore, the pharmacist's professional growth appeared to extend beyond the confines of the research study.

While the training, mentorship and competency assessment might appear resource intensive for a six-month intervention, many PIPs reported benefits beyond the study. In future, there is a need to consider a training approach which prepares pharmacists not only with clinical skills but also with higher level management skills to support relationship building across new teams.

## Supplementary Information

**Additional file 1:** **Supplementary file 1.** Service Specification and how covered in process evaluation

**Additional file 2:** **Supplementary file 2.** Care homes independent pharmacist prescribing study (chipps) training programme

**Additional file 3:** **Supplementary file 3.** CHIPPS WP6 QUAL Evaluation: Topic guide for PIP

**Additional file 4:** **Supplementary file 4.** Demographic details of triads and participants in CHIPPS study

**Additional file 5:** **Supplementary file 5.** PIPs evaluation of whether a topic should be covered, if session delivered effectively and if time allocation was sufficient

## Data Availability

The datasets used and/or analysed during the current study are available from the corresponding author on reasonable request.

## Abbreviations

GP: General Practitioner
IP: Independent Prescriber
PCP: Pharmaceutical Care Plan
PIP: Pharmacist Independent Prescriber
