# Supplementary material for: Evaluation of a training programme for Pharmacist Independent Prescribers in a care home medicine management intervention
Source: BMC Med Educ. 2022 Jul 15;22:551. doi: 10.1186/s12909-022-03575-5 (PMC9287970; doi:10.1186/s12909-022-03575-5)
Supplement: Supplementary file 1 — Additional file 1: Supplementary file 1. Service Specification and how covered in process evaluation [file 12909_2022_3575_MOESM1_ESM.pdf]

Supplementary file 1 Service Specification and how covered in process evaluation

**Recruitment and employment of the Pharmacist Independent Prescriber (PIP)**	
Specification	*Implementation*
Excellent interpersonal, communication and IT skills	No PIP communication problems reported<br><br>Two PIP report challenges with communication with some Care home staff (CHS)
Familiarity with relevant GP software systems	No data
Experience of providing prescribing and medicines management advice and support	Strong evidence from PIP and CHS
Previous experience of working in GP practice environment	Variety of experience on personal CVs<br><br>Difference in experience of implementation related to previous employment in practice rather than PIP experience
Be able to travel to site locations	Not identified as a problem, some could walk 137 hours logged as travel activity.<br><br>Multiple CH more difficult to manage in 4 hours a week
A mobile phone to be contactable for the purposes of delivering this service	Data not collected but 1 PIP said left mobile number
Appropriate indemnity insurance for prescribing	Held with Norfolk and Waveney CCG
**Review each resident's medication and develop and implement a pharmaceutical**	
Specification	*Implementation*
Optimise prescribing ensuring clear indication and evidence base for each medication (taking into consideration national and local pathways, guidelines and formularies), informed by tools such as STOPP/START	Process evaluation data did not cover this detail<br><br>Very few concerns raised during PCP review<br><br>No concerns for 22 PIP during clinician review
**Care plan (essential)**	

Specification	*Implementation*
Minimise the potential for adverse effects	As above
Optimise the dose of all medication	In study pharmacist review<br><br>18 (17.5%) PCPs for which the missed opportunity had a theoretical link to falls risk; these were all lack of a documented review of two or more medications with anticholinergic effect. Of these, two were considered a definite falls risk, whilst sixteen were considered possible<br><br>CH reported easy access to PIP enabled drug doses to be monitored and changed as required
Co-ordinate appropriate monitoring and associated tests for all medicines and conditions	In Study pharmacist review:<br><br>30 (29.1%) PCPs had missed opportunities for investigational monitoring<br><br>16 (15.5%) PCPs had missed opportunities for clinical monitoring
Agree initial care plan with GP, care staff and resident (where appropriate)	Formal agreement of care plan not mentioned during interviews by any person
Document and maintain records relating to review and care plan in GP and care home records as appropriate	PCP review by study pharmacists identified<br><br>38 PCPs (36.2%) had no comments,<br><br>42 PCPs (40.7%) had one or more medications for which there was no documented indication<br><br>5 PCPs (4.8%) had a comment regarding either the dosage of one or more medication or the duration of therapy
**Prescribing (essential)**	
Specification	*Implementation*
Authorise repeat prescriptions	Survey indicates PIP varied in reauthorizing prescriptions:<br><br>10 PIPs reauthorised all residents<br>2 PIPs reauthorised most residents<br>1PIP reauthorised some residents

	2 PIPs reauthorised a few residents<br>1 PIP reauthorised no residents<br><br>Some PIPs stated due to limited time of intervention not worth changing reauthorising procedures<br><br>Of the 8 GP survey responses 3 stated PIPs did some repeat prescriptions remainder say none or don't know
Co-ordinate appropriate monitoring and associated tests for all medicines and conditions	Generally, GP found this helpful<br><br>Two GPs disagreed with PIP decisions
Deprescribe medicines according to agreed pharmaceutical care plan	This was evident throughout the data
Document medication changes in GP and care home records and notify supplying pharmacy of all changes to medication within 24 hours	PCP study pharmacist review found some document missing.<br><br>Qualitatively PIPs said finding time to fully complete PCP was a challenge
Only initiate new medicines for existing diagnoses or for common ailments which can be managed with medicines classified by the Medicines and Healthcare products Regulatory Agency (MHRA) as Pharmacy (P) or General Sales List (GSL)	No data
Any additional areas of prescribing must be agreed and documented with the GP practice prior to prescribing (e.g. antibiotics for simple UTIs)	No data, but evidence that PIPs referred to GP if unsure of their competence or decision
**Communication (essential)**	
Specification	*Implementation*
Agree local protocols for communication with GP practice and care home prior to commencing service. This should include:<br><br>• Process of communication and messaging<br>• The location and expected level of detail of all PIP interventions in the medical records	Communication with GP generally through computerised 'Task' system<br><br>Face to face meeting were not generally formalised and finding time to meet with GP could be a challenge for some. This was more likely when PIP not employed in surgery

• Process and communication of referrals for activities outside the competence of the PIP	PIPs spoke of agreeing time to visit CH with manager, not just dropping in, but changes in staff created challenges with continuity and the 'busyness' of CH meant not always someone free to support PIPs access to residents or notes
Inform supplying community pharmacy about service and role prior to commencing service)<br><br>• Communicate all changes in medication to supplying pharmacy	All PIPs reported working with Community Pharmacist and it seemed close working relationships developed in most setting
Complete all documentation and recording of activities as required by the study team.	Generally yes, but some PIPs stated not record all activity and delays in completing PCPs even after their phase had ended
**Support systematic ordering, prescribing, and administration processes with each care home, GP practice and supplying pharmacy where needed (at PIP's discretion)**	
Specification	*Implementation*
Provide instructions on how to administer each drug	No data
Synchronise residents' prescription quantities for monthly cycles	Some PIPs very alert to this to avoid over ordering
Add or clarify directions for all medication where it is currently not clear	No data
Provide advice on repeat prescription ordering processes to:<br><br>• Minimise missed items<br>• Optimise quantities	Took several PRN drugs of repeat prescriptions. Several PIPs said CCG pharmacist or others had visited and prescribing and ordering systems robust
Support optimal the use of homely remedies within the care home	8 PIPs reported this activity in survey<br><br>A couple of PIPs moved rarely used PRN drugs to homely remedies. A couple offered training and help on policies but not generally a large part of activity
Reconcile resident medication following a transfer of care	Not reported in qualitative data but acknowledged as important part of role and one incident post intervention of PIP recognising secondary care error
**Training provision (undertaken at PIP's discretion)**	
Specification	*Implementation*

Review training needs of care home and GP practice and draft proposed training plan	No evidence of formal review of training needs
Provide training to care home staff on needs basis from agreed list of potential topics/areas	Few did this: 2 PIPs stated once and 4 PIPs stated more than once.<br><br>Reasons for not doing it were other training in place and lack of time
Provide guidance to relevant GP practice on training needs basis from agreed list of potential topics/areas	
**Safe and effective service provision**	
Specification	*Implementation*
PIP will be contactable and respond to messages within 24 hours (Monday - Friday)	Some PIPs worked part time so this not possible
The PIP will establish a locally agreed protocol with the GP practice for referral/notification of all medicine related queries from CHIPPS participants to the PIP as appropriate	PIP Received protocol to refer medication queries to GP — 10 — 59%
PIP will have full (read/write) access to GP record system to issue prescriptions and update records	PIP Had read access to GP/care home records — 16 — 94%<br>PIP Had write access to GP/Care Home records — 15 — 88%<br>PIP Had remote access to GP/Care home records — 7 — 41%
Where possible, PIP will use remote access to update records when changes are made to GP held record<br><br>• Where remote access is not feasible the PIP must update records within 24 hours of making a change	See above
PIP will have full (read/write) access to care home records to update records during all visits using appropriate local reporting systems	See above
The PIP will visit/contact the care home at least once a week	8 PIPs visited monthly<br><br>9 PIPs visited weekly

	From survey data. In some triads communication between PIP and CH was remote, by phone or email. For some visits reduced during the intervention. Activity log indicates most time spent in desk based resident activity
The PIP will visit/contact the GP practice at least once a week	1 PIP visited monthly<br>3 PIPs visited weekly<br>11 PIPs visited daily
Wherever possible, all annual leave should be agreed before the beginning of the study. A clear system for transfer of responsibility communicated to GP, care home and supplying pharmacy	No data
The PIP will work within the local prescribing formularies of GP practice and primary care organisation	No data
The PIP will report and document all significant clinical events or near misses using local reporting procedures and study documentation.	Yes, clear reporting to CTU
Ensure all records are aligned	No data
