# Supplementary material for: Evaluation of a training programme for Pharmacist Independent Prescribers in a care home medicine management intervention
Source: BMC Med Educ. 2022 Jul 15;22:551. doi: 10.1186/s12909-022-03575-5 (PMC9287970; doi:10.1186/s12909-022-03575-5)
Supplement: Supplementary file 2 — Additional file 2: Supplementary file 2. Care homes independent pharmacist prescribing study (chipps) training programme [file 12909_2022_3575_MOESM2_ESM.pdf]

Care Homes Independent Pharmacist Prescribing Study (CHIPPS)

Training programme

1 Overview

Pharmacist Independent prescriber (PIP) competency development for CHIPPS will consist of the following:

- Training days (Study design & project delivery, preparation for role)
- Development of underpinning knowledge
- Assessment against competency framework and agreement of personal development plan with mentor
- Relationship building and logistics sessions (4 days)
- Sign off for role through competency panel consisting of doctor and pharmacist

NB: This training will be in addition to the 16 hours a month in Section 5.

2 Training days

Day 1: Study design and project delivery

- Trial rationale, design & outcomes (RH) 2 hours
- Training and development plan (DW) 2 hours
    - Personal development planning and sign off
    - Provision of guidelines to support underpinning knowledge
    - Relationship building and logistics sessions
- Effective communication and record keeping (include care planning) 1 hour
- Service initiation & implementation (Develop protocol) (DW) 2 hours

Day 2: Preparation for role

- Managing the frail elderly complex person (Consultant geriatrician) 5 hours
    - Case studies (show STOPP/START)
    - Identifying boundaries i.e. red flags which require referral
    - Anticholinergic burden, antipsychotic de-prescribing
    - Assessing capacity and gaining consent (covert administration, family involvement)
- Medicines management (Error management, efficient prescribing) 2 hours

3 Underpinning knowledge

Specific knowledge required to practise safely in a care home:

Conditions
- Parkinson's disease
- Cognitive impairment and behavioural disturbances

Symptoms
- Delirium
- Common skin conditions seen in care homes
- Dysphagia

Non-pharmacological therapy
- Wound management & catheter and stoma prescribing guidelines*
- Nutrition guidelines*
- Pain
- Dose optimisation based on renal function

Pharmacological therapy
- Cardiovascular (Hypertension, Secondary prevention, Heart failure)
- Asthma & COPD
- Anti-coagulant
- Anticholinergics & burden
- Antipsychotic
- Sedatives
- Antidepressants
- GI (Laxatives, PPIs)
- Diabetes

Legislation
- Mental Capacity Act or local equivalent and gaining consent
- Covert administration
- Controlled drugs

Signposting
- Direct to other health care professionals as required

Safety
- As a registered pharmacist, in accordance with standard practice and their professional code of Ethics, the PIP will be professionally obliged to take swift and proportionate action if they observe any practices by colleagues which raise concerns.
- In all circumstances local NHS procedures and policies would be followed. Additionally any such concerns would be reported in confidence to the Principal Investigators. In all cases a written record of any incidents and subsequent action taken will be held securely with access restricted to relevant study team members and we will incorporate guidance on this process within the PIP training package.

* Local guidelines

4 Independent prescribing in care homes competency framework

Domain	Competency	Behaviours
Prescribing	Safe and effective therapy alteration	• Discontinues or changes therapy in line with best practice<br>• Implements appropriate monitoring plans for safety and efficacy for medicines which have been initiated, changed or discontinued
	Effective monitoring of therapy	• Implements monitoring according to local requirements and expectations<br>• Ensures that prescribing and monitoring practices relating to high risk therapy e.g. anti-platelet and anticoagulant therapy, are appropriate
	Recognises limitations of competence	• Identifies complex prescribing decisions outside of competency and seeks appropriate support and guidance<br>• Identifies where patients transfer from chronic disease management to terminal care and hands responsibility back to medical practitioner
Medicines Management	Medicines storage, handling and record keeping	• Supports the home in meeting national requirements for safe medicines management<br>• Identifies and recommends practical solutions to improve medicines related activities e.g. addresses inequivalence, reduces wastage and likelihood of medication related errors<br>• Recognises advantages and disadvantage of medication administration systems and identifies approaches to optimise their use
	Medicines reconciliation	• Supports effective transfer of medicines related information when residents are hospitalised<br>• Ensures that medicines related information transferred from hospital to the care home is accurate and complete
	Safe and effective medicines administration	• Ensures that care staff know how to administer medicines safely and appropriately e.g. when it is appropriate to crush/disperse medicines<br>• Identifies patients for whom administration of medicines is challenging and supports care staff accordingly
	Responds appropriately to medicines related errors and critical incidents	• Performs critical incident analysis, identifying and implementing strategies to prevent future recurrence<br>• Supports recording and reporting of incidents in line with local and national policy
Communication	Maintenance of records related to prescribing and medication review	• Uses IT systems within care home and general practice effectively<br>• Ensures all activities and rationale for them are communicated effectively and recorded contemporaneously

		• Ensures records of activities are accessible to care home staff and members of healthcare professional team<br>• Language used within records appropriate for all stakeholders
	Relationship building and maintenance	• Appropriately uses and refers to all members of healthcare team responsible for care within the home<br>• Whenever practical and appropriate involves residents, families and carers in prescribing decisions<br>• Regularly reviews role and boundaries to maintain effective relationship
	Trains others	• Delivers effective small group teaching sessions<br>• Provides feedback on performance sensitively and constructively
Managing complexity in later life	Pain management	• Recognises the symptoms associated with pain in patients with and without cognitive impairment<br>• Ensures that 'as required' pain relief is supplied when necessary e.g. paracetamol
	Cognitive impairment (5)	• Regularly reviews all antipsychotic, sedative and anticholinergic therapy for need and appropriateness
	Nutrition	• Ensures that resident nutritional needs are regularly reviewed and related prescribing is in line with local policy and guidance<br>• Ensures that appropriate nutritional support is provided to enhance bone protection
	Polypharmacy	• Reviews and rationalises therapy in light of risk and benefits in a complex older person<br>• Appropriately reviews therapies which are known to increase the likelihood of falls
Context	Cultural awareness	• Practices in line with expectations of general practitioners with respect to inter-professional working and prescribing practices<br>• Identifies medicines related cultures (e.g. use of antipsychotics, sedatives, antibiotics, analgesia & laxatives) within the care homes and works with staff to implement best practice<br>• Educates and supports care staff in the management of behavioural disturbances and the use of antipsychotic medication
	Policy awareness	• Supports home in meeting relevant legislative frameworks e.g. CQC requirements<br>• Ensures patient rights under mental capacity act e.g. covert administration, right of refusal
	Duty of candour	• Appropriately acts and reports on practices which fall below expected standards

5 Relationship building and logistics sessions

All of the following should be completed in 2 - 4 hour slots

Develop relationship with general practitioner and medical practice 16 hours

- Discusses and agrees prescribing boundaries and methods of working with general practitioner
- Identifies efficient approaches to manage urgent medicine requests from home
- Agrees frequency and preferred modes of communication with general practitioner
- Visits care home with GP when undertaking a routine visit and jointly perform medication review on non-study residents
- Identifies support available for the home from other healthcare professionals and how to refer residents to them
- Develops list of useful contacts
- Learns how to use IT system and agrees content of records
- Obtains access to prescription pad
- Meets lead prescriber and local primary care pharmacist responsible for prescribing within medical practice to obtain local medicines related problems and policies

Develop relationship with care home 8 hours

- Discusses and agrees role and boundaries associated with this within care home
- Agrees frequency and preferred modes of communication with care home
- Discusses care plans, completion, updates and implementation
- Understands recording systems including MAR charts
- Observe medication administration rounds for recruited residents to identify how best to support staff
- Identify senior members of team, roles and medicines related culture
- Identifies other healthcare professionals associated with the home

Proposed care home staff training (6x30 minute sessions) (Part of service delivery)

For all homes:

- Outline of PIP role

Delivered as per identified home needs:

- Medicines administration
- Antipsychotic medicines
- Antibiotics
- Constipation and laxative use
- Pain management

All packs obtained or prepared for the PIPs by the CHIPPS team for standardisation purposes

Develop relationship with community pharmacist **4 hours**

- Meets local community pharmacist who supplies home to agree methods of working
- Identify date for prescription requisition and for medicines supply to home
- Understands medicines supply system used and relative advantages and disadvantages
- Identifies any process related issues which they can assist with
- Agrees preferred methods of communication

Understanding context more broadly **4 hours**

Arrange to meet the following if available and deemed useful:

- Local safety expert i.e. person responsible for safety from medical practice perspective
- Care home pharmacist specialist
- Consultant geriatrician working in primary care
- Community matron with responsibilities for care homes
- Local CQC or national equivalent inspector
- District nurse (Dressings, catheter, barrier cream policies)

6 Personal development planning and sign-off

Each PIP will be assigned a pharmacist mentor who will be a local expert in care homes medicines.

They will use the underpinning knowledge and competency framework to agree:

- A personal development plan
- Evidence to be collated
- Time plan for completion.

Once the training is completed each PIP will meet up with a local expert medical practitioner (assessor) and their mentor to review their progress against the personal development plan.

The outcomes of the process will be either:

- Sign off to participate in the study
- Sign off of the pharmacist to participate in the study and identification of additional training needs which can be safely developed as the service is implemented.
- Non-sign off and agreement to either collect more evidence and represent it or withdraw from the study.
