# Supplementary material for: Evaluation of a training programme for Pharmacist Independent Prescribers in a care home medicine management intervention
Source: BMC Med Educ. 2022 Jul 15;22:551. doi: 10.1186/s12909-022-03575-5 (PMC9287970; doi:10.1186/s12909-022-03575-5)
Supplement: Supplementary file 3 — Additional file 3: Supplementary file 3. CHIPPS WP6 QUAL Evaluation: Topic guide for PIP [file 12909_2022_3575_MOESM3_ESM.pdf]

CHIPPS WP6 QUAL Evaluation: Topic Guide for PIP

As the purpose of this interview is to encourage a conversation in which the participant can feel confident in expressing their own views, the following topic guide is indicative. This means that the interviewer may adapt it to suit the conversation style and preferences of the participant.

	**<u>Introduction.</u>**<br>**1. Introduce yourself**<br>**2. Explain the purpose of the research**	We have developed a new service in which a specially trained 'pharmacist independent prescriber' (PIP) becomes part of the care home team, working alongside general practitioners. The aim is to improve how resident's medicines are managed in a safe, effective and cost effective way. We hope this may improve resident's care, wellbeing and health outcomes. We are evaluating this new service in a large multi-centre randomised controlled trial in different places in the United Kingdom.<br>The study is run by the universities of East Anglia, Aberdeen, Leeds and Queen's Belfast. It is funded by the UK National Institute for Health Research.<br>*Since participating in the training for 'pharmacist independent prescribers (PIPs) ' you have set up and have delivered the Care Home Independent Pharmacists Prescriber service in the trial,*<br>*We would like you to consider your overall role as a PIP providing this service and your experience in delivering this. We would also like your views on the Service Specification and Pharmaceutical Care Plan, your thoughts about the mentoring you received and how useful the personal development plan and training was in preparing you for this role and how this has impacted on your ability to set up and deliver this service.*
	**<u>The digital recorder</u>**<br>**1. Stress confidentiality**	I would like to highlight the confidentiality of everything you tell me, and specifically that:<br>• *the recording will be deleted after being transcribed*<br>• *you won't be identified individually in any report.*<br>• *all information will be anonymised.*<br>• *we will not tell anyone else including your employer organisation, what you tell us as an individual.*<br>• *likewise no other individual e.g. GP, care home staff, resident will be identifiable in any report.*

	**2. Set ground rules**	*We do need to remind you, however, that* if you do disclose anything which might identify a risk to yourself or to others, or a personal or professional offence, this would be shared with the relevant responsible authority. However we would tell you if we thought this were the case.<br><br>• All of your views are of value to us. There are no right or wrong answers,<br>• Please ask me to clarify if the question isn't clear.<br>• We remind you not to share any personal or patient identifying information during this interview
	**Ask if there are any questions**	
	**Confirm consent**	

	**Stem question PIP service delivery**	**Probes / follow ups**
1.	What was your experience of delivering the service?	• Any particular issues<br>• Anything particularly good<br>(Interaction with GP/ care home staff/residents/ relatives access to records/ information/routines /types of interventions)<br>• Acceptability to residents without capacity
2	In what ways did your service affect resident care	• Examples positive and negative
3.	What are your views on the Service Specification?	• Clarity?<br>• Level of detail?<br>• Inclusion/exclusion criteria<br>• Service requirements?<br>• Outcomes<br>• Transitional arrangements
	Stem Question –research activity	•
34	What are your views on the research study documentation including: Pharmaceutical Care Plan, PIP log and resource use data?	• Ease of use<br>• Anything missing/ not needed<br>• Fit for purpose?

		• How much time did it take
		•
5.	What were your thoughts on the role of the mentor?	• Positive<br>• Negative<br>• Areas for improvements
6.	What was your experience of developing your own personal development plan with your mentor?	• Went well/ not so well<br>• Mentor support/ time scale for completion<br>• Accessing training on the identified development areas<br>• General acceptability
7.	What do you think about the competency framework assessment?	• Comprehensive?<br>• Easy/hard to demonstrate achievement?
78	What do you think about the assessor signing you off?	• Useful / difficult discussion?<br>• Appropriate?<br>• Alternative suggestions?<br>• Process fit for purpose?
	**Stem question Training**	**Probes / follow ups**
89	What are your thoughts on the impact of training you received?	Any gaps-<br>• *preparation for role,*<br>• *underpinning knowledge,*<br>• *competency framework,*<br>• *relationship building*<br>• *personal development skills*
10.	Now that you have been delivering the service for 3 months which particular elements of the training do you think have been most useful?	• Why?<br>• Relevance<br>• Time spent on this<br>• Delivery
11.	Which particular elements of the training do you think have been least useful in your role, delivering the service?	• Should we continue to provide this element of the training?

		• How should this element of training be delivered?<br>• What should we do to improve these elements of training?
11.	Is there anything that should have been included in the training that wasn't?	• How would you suggest that we train pharmacists in that?<br>• Is there existing training available, that you are aware of, to meet this<br>• Need?<br>• When should this training be provided?
12.	It is important to us that across the four areas, all PIPs provide a similar service. What would be the best way for this to be achieved?	• Training?<br>• Checklist?
13.	In what ways could you contribute to the training of the next cohort of Pharmacist Independent Prescribers?	• Mentor?<br>• Helping to deliver educational package locally?
14	How important for you was peer support	• use The Telegram group<br>• Use other ways if staying in contact
	**Stem question on team work**	•
8.	Where did communication issues arise between your and the other team members involved in the PIP service?	• Care homes<br>• Other Pharmacist<br>• Primary care team<br>• GP Practice<br>• Relatives/residents
9.	How do you think communication impacted on resident care? (positively/ negatively)	• Examples
14.	Any final comments?	
15.	Thank you for taking part in this interview	

N.B. all interview topic guides started with the setting the introduction and digital record prompts; in the remainder of the document we only include stem questions.

CHIPPS WP6 QUAL Evaluation: Topic Guide for GP

	**Stem question**	**Probes / follow ups**
Part 1	Intervention	
1.	Overall what are your views of the PIP service?	• New contributions<br>• New problems
	Views on changing skill mix in the primary care team<br>Views on changing skills mix in the care home team	• Diversity in team(s)<br>• Challenges<br>• Issues in managing
2.	How has the PIP service impacted on your work load?	• (Negatively/ positively)<br>• Time<br>• Visit frequency<br>• Referrals<br>• Tests<br>• Medication changes<br>• Medication reviews<br>• Medication ordering<br>• Repeat prescriptions
3.	In what ways has the PIP service impacted on patient care?	• Examples<br>• Acceptability for patients without capacity?
Part 2	Implementation	
4.	What barriers if any to implementing this service?	
	How could we overcome any barriers?	
5.	What did you see as facilitating the service to be implemented?	

	Stem question	Probes / follow ups
	**Stem question**	**Probes / follow ups**
Part 3	Working relationship	
6.	What is your view of your working relationship with the PIP?	• Good relationship areas<br>• Difficult relationship areas
7.	Could the working relationship with the PIP have been improved?	
8.	How has the new service affected your relationship with your patients?	• New contributions<br>• New problems
9.	How has the new service affected your relationship with the care home staff?	• New contributions<br>• New problems
10.	Where did communication issues arise between you and the other team members of the team involved in the PIP service, and what were they?	• PIP<br>• Care Home<br>• Community Pharmacist<br>• Primary care Pharmacist<br>• District Nurse<br>• GP Practice
11.	How do you think any communication issues may have affected patient care?	• (Positively/ negatively)<br>• examples
Part 4	Acceptability	
12.	What aspects of the PIP service went well from your perspective?	• Time commitment<br>• Paper work<br>• Issues raised/resolved
13.	What aspects of the PIP service went less well from your perspective?	• Time commitment<br>• Paper work

	Stem question	Probes / follow ups
		• Issues raised/resolved
	How could we improve these?	
14.	What elements of the service did you like best?	• Patient care
15.	Overall, how satisfied are you with the service provided by the PIP?	
16.	Would you like the service to continue?	• In its current from<br>• In a revised form<br>• Not at all
17.	Any final comments?	
18.	Thank you for taking part in this interview	

CHIPPS WP6 QUAL Evaluation: Topic Guide for Care Home Manager

	Stem question	Probes / follow ups
Part 1	Views of the intervention	
1.	Overall what are your views of the PIP service?	• New contributions<br>• New problems
	Views on changing skill mix in the primary care team<br>Views on changing skills mix in the care home team	• Diversity in team(s)<br>• Challenges<br>• Issues in managing
2.	How has the PIP service impacted on your work load? (positively/negatively)	• Time<br>• Referrals<br>• Tests<br>• Medication changes<br>• Medication reviews<br>• Repeat prescriptions<br>• Medication administration processes<br>• Record keeping<br>• Medication storage<br>• Interactions with GPs<br>• Interaction with community pharmacist<br>• Number of pharmaceutical advisory visits (i.e. not including PIP)
3.	In what ways has the PIP service affected patient care? positively/negatively)	• Examples<br>• Acceptability for patients without capacity?

	**Stem question**	**Probes / follow ups**
4.	Have you or your staff received any additional training since the introduction of the PIP service?	• Impact?
Part 2	Implementation	
5.	What barriers if any to implementing this service?	
	How could we overcome any barriers?	
6.	What did you see as facilitating the service to be implemented?	
Part 3	Working relationship	
7.	What is your view of your working relationship with the PIP?	• Good relationship areas<br>• Difficult relationship areas
	How can your working relationship with the PIP be improved?	
8.	What is your view of your staffs' relationship with the PIP?	• Good relationship areas<br>• Difficult relationship areas
	How can their working relationship with the PIP be improved?	
9.	How has your working relationship with the PIP affected your relationship with your patients?	• New contributions<br>New problems
10.	Where did communication issues arise between you and the other team members involved in the PIP service, and what were they?	• PIP<br>• Community Pharmacist<br>• Primary care Pharmacist<br>• District Nurse<br>• GP Practice
11.	How do you think communication issues may have affected patient care?	• Examples
Part 4	Acceptability	
12.	What aspects of the PIP service went well from your perspective?	• Time commitment<br>• Paper work<br>• Issues raised/resolved
13.	What aspects of the PIP service went less well from your perspective?	• Time commitment

	**Stem question**	**Probes / follow ups**
		• Paper work<br>• Issues raised/resolved
	How could we improve these?	
14.	What elements of the service did you like best?	
15.	Overall, how satisfied are you with the service provided by the PIP?	
16.	Would you like the service to continue?	• In its current from<br>• In a revised form<br>• Not at all
Part 5	Study procedures	
17.	Recruitment and participation	• Acceptability for residents with/without capacity<br>• Time commitment<br>• Paper work
18.	Any final comments	
19.	Thank you for taking part in this interview	

CHIPPS Care Homes Independent Pharmacist Prescribing Study

CHIPPS WP6 QUAL Evaluation: Topic Guide for Care Home Staff

	**Stem question**	**Probes / follow ups**
Part 1	Views of the intervention	
1.	What involvement have you had with the PIP service?	
2.	Overall what are your views of the PIP service?	• New contributions<br>• New problems
	Views on changing skill mix in the primary care team<br>Views on changing skills mix in the care home team	• Diversity in team(s)<br>• Challenges<br>• Issues in managing
3.	How has the PIP service impacted on your work load? (positively/negatively)	• Time<br>• Referrals<br>• Tests<br>• Medication changes<br>• Medication reviews<br>• Repeat prescriptions<br>• Medication administration processes<br>• Record keeping<br>• Medication storage<br>• Interactions with GPs<br>• Interaction with community pharmacist
4.	In what ways has the PIP service affected patient care? positively/negatively)	• Examples<br>• Acceptability for patients without capacity?
5.	Have you received any additional training since the introduction of the PIP service?	• Impact?
Part 2	Implementation	

	Stem question	Probes / follow ups
6.	What barriers if any to implementing this service?	
	How could we overcome any barriers?	
7.	What did you see as facilitating the service to be implemented?	
Part 3	Working relationship	
8.	What is your view of your working relationship with the PIP?	• Good relationship areas<br>• Difficult relationship areas
9.	*How can your working relationship with the PIP be improved?*	
10.	How has your working relationship with the PIP affected your relationship with your patients?	• New contributions<br>New problems
11.	Where did communication issues arise between you and the other team members involved in the PIP service, and what were they?	• PIP<br>• Community Pharmacist<br>• Primary care Pharmacist<br>• District Nurse<br>• GP Practice
12.	How do you think communication may have affected patient care? (positively/ negatively)	• Examples
Part 4.	Acceptability	
13.	What aspects of the PIP service went well from your perspective?	• Time commitment<br>• Paper work<br>• Issues raised/resolved
14.	What aspects of the PIP service went less well from your perspective?	• Time commitment<br>• Paper work<br>• Issues raised/resolved
	How could we improve these?	
15.	What elements of the service did you like best?	• Patient care

	**Stem question**	**Probes / follow ups**
16.	Overall, how satisfied are you with the service provided by the PIP?	
17.	Would you like the service to continue?	• In its current from<br>• In a revised form<br>• Not at all
18.	Any final comments?	
19.	Thank you for taking part in this interview	

CHIPPS Care Homes Independent Pharmacist Prescribing Study

CHIPPS WP6 QUAL Evaluation: Topic guide for residents or relatives

	**Stem question Resident/relatives**	**Probes / follow ups**
Part 1 Awareness of the intervention		
1.	Overall have you noticed any changes in your care/ the care of your relative/friend since the introduction of the PIP service?	
	What changes have you noticed?	• Good/Bad<br>• Examples
Part 2 Implementation		
2.	Before the PIP service started, what did you think about the information you received describing the new service?	• Relevant to you<br>• Amount of detail<br>• Understandable
3.	Given information you had before the PIP service started, how far was the service what you thought it would be?	• Examples
Part 3 Relationship with PIP		
4.	How did you get on with your PIP?	• Good relationship areas<br>• Difficult relationship areas
	How could your relationship with the PIP be improved?	
5.	How easy did you find it to talk to the PIP?	• Examples
6.	How do you think your relationship with the PIP affect your care/ your relative/friends care?	• Examples
Part 4 Acceptability		
7.	What aspects of the PIP service went well from your perspective?	• Examples
8.	What aspects of the PIP service went less well from your perspective?	• Examples
9.	Is there anything we could improve the overall service?	• Examples
10.	Overall how satisfied are you with the service provided by the PIP?	

Part 5	Study procedures	
16.	Recruitment and participation	• Acceptability for residents without capacity
11.	Any final comments?	
12.	Thank you for taking part in this interview	

End document
