# Supplementary material for: Evaluation of a training programme for Pharmacist Independent Prescribers in a care home medicine management intervention
Source: BMC Med Educ. 2022 Jul 15;22:551. doi: 10.1186/s12909-022-03575-5 (PMC9287970; doi:10.1186/s12909-022-03575-5)
Supplement: Supplementary file 4 — Additional file 4: Supplementary file 4. Demographic details of triads and participants in CHIPPS study [file 12909_2022_3575_MOESM4_ESM.pdf]

Additional file 3 Demographic details of triads and participants in CHIPPS study

Triad ID	PIP: Yr pharmacist	PIP: Yr~ Independent prescriber	PIP: CH experience	PIP: No. residents allocated	PIP: Activity~ hrs	GP: Practice size < or ≥10K	GP: Location	GP: IMD	GP: GP employs PIP	Care home 1: CH type	Care home 1: Funded	Care home 1: IMD	Care home 1: No. CH beds	Care home 1: Changed managers	Care home 2: CH type	Care home 2: Funded	Care home 2: IMD	Care home 2: No. CH beds	Care home 2: Changed managers	Care home 3: CH type	Care home 3: Funded	Care home 3: IMD	Care home 3: No. CH Beds	Care home 3: Changed managers	Data collection – Interviews: PIP	Interviews: GP	Interviews: CHM	Interviews: CHS	Questionnaire: PIP	Questionnaire: GP	Questionnaire: CHM
1	12	1month	N	6	50	<	urban	3	Y	dual	pri	10	33	0											*		*		*	*	
2	21	2months	Y	20	86	≥	semirural	8	Y	dual	pri	8	60	0	dual	pri	8	38	0						*				*		
3	12	1	y	16	69	<	urban	6	N	res	LA	7	29	0	res	LA	6	35	0							*					
4	20	1	N	24	12	≥	urban	10	N	dual	pri	10	60	0	res	pri	10	34	0						*				*		
5	9	1 1/2	Y	20	88	<	urban	1	N	res	LA	3	89	1																	
6	10	1 1/3	N	22	48	≥	urban	2	N	res	Vol	5	44	0											*	*	*	*	*	*	*
7	23	2	N	9	44	<	rural	8	Y	dual	pri	8	45	0																*	
8	24	1 1/2	Y	24	70	≥	semirural	4	Y	dual	pri	4	29	0	res	pri	8	34	0						*	*		*	*		
9	25	1 1/2	N	21	92	≥	rural	3	Y	res	pri	6	62	0											*		*		*		
10	10	2	N	23	84	≥	urban	4	Y	dual	pri	7	60	0																	
11	30	3	N	14	78	<	semirural	10	Y	res	pri	8	33	0											*	*		*	*		
12	25	4	y	19	68	≥	Urban	10	y	res	Vol	5	84	0	res	pri	10	44	0	res	Vol	10	46	0			*				
13	8	4	N	20	145	≥	urban	4	N	dual	pri	2	83	1															*	*	
14	12	4	N	20	34	<	rural	9	Y	dual	pri	4	33	0	dual	pri	9	27	1	dual	pri	9	18	1	*	*	* *	*	*		
15	10	6	99	18	42	<	urban	1	Y	dual	pri	7	65	3											*				*	*	
16	11	6	Y	20	107	<	urban	1	N	dual	pri	1	76	1											*	*				*	
17	16	7	Y	6	20	missing	urban	3	Y	dual	pri	7	66	0	dual	pri	10	58	0	dual	pri	10	29	0	*				*		
18	31	8	N	9	15	<	urban	4	Y	dual	pri	6	41	0	dual	pri	8	57	1								*		*		
19	32	9	Y	24	99	<	rural	6	N	res	pri	6	47	0											*	*		*	*	*	
20	30	10	Y	23	60	missing	semirural	5	Y	dual	pri	7	48	0	dual	pri	8	64	0	dual	pri	5	35	0	*				*		
21	40	14	Y	21	45	<	urban	10	y	dual	pri	10	35	0	dual	pri	6	41	0							*	* *	*	*		*
22	36	16	Y	11	50	<	urban	10	Y	dual	pri	5	53	1											*				*		
W/D	18	1	N	11	missing	<	urban	3	Y	dual	pri	2	40	1																	
W/D	8	2	N	24	missing	≥	urban	6	N	res	pri	3	28	0	res	pri	5	24	0											*	
W/D	5	1 1/2	N	24	missing	≥	urban	3	N	res	pri	1	98	0																	

Key

CH Care home

IMD Indices of multiple deprivation
