# Supplementary material for: Evaluation of a training programme for Pharmacist Independent Prescribers in a care home medicine management intervention
Source: BMC Med Educ. 2022 Jul 15;22:551. doi: 10.1186/s12909-022-03575-5 (PMC9287970; doi:10.1186/s12909-022-03575-5)
Supplement: Supplementary file 5 — Additional file 5: Supplementary file 5. PIPs evaluation of whether a topic should be covered, if session delivered effectively and if time allocation was sufficient [file 12909_2022_3575_MOESM5_ESM.pdf]

Supplementary file 5 PIPs evaluation of whether a topic should be covered, if session delivered effectively and if time allocation was sufficient

**Topic should be covered**	**Strongly disagree**	**Disagree**	**Unsure**	**Agree**	**Strongly agree**
**Clinical areas**					
Training Plan				4	21
Pharmaceutical care planning				4	21
Efficient Prescribing and error management **A** n=16			2	3	11
Antipsychotic medicines in care homes				2	23
Covert administration and dysphagia				4	21
Care home Medicine **B** N=15			3	3	9
Complex patient case studies				5	20
PIP Case studies				3	22
**Research procedures**					
CHIPPS overview			1	4	20
Research issues & practical procedures				4	21
CHIPPS trial Internal Pilot and RCT				7	18
Capacity assessment and family involvement **C** n=10			1	1	8
Mentor meeting **D** N=10				1	9
Next steps E n =18			1	1	20

Session effectively delivered

**Session effectively delivered**	**Strongly disagree**	**Disagree**	**Unsure**	**Agree**	**Strongly agree**
**Clinical areas**					
Training Plan				4	21
Pharmaceutical care planning		1	2	6	16
Efficient Prescribing and error management **A** n=16		1	1	5	9
Antipsychotic medicines in care homes			1	5	19
Covert administration and dysphagia			1	3	21
Care home Medicine **B** N=15			3	3	9
Complex patient case studies				6	19
PIP Case studies			1	5	19
**Research procedures**					
CHIPPS overview			1	5	19

Research issues & practical procedures			3	4	18
CHIPPS trial Internal Pilot and RCT				7	18
Capacity assessment and family involvement **C** n=10			1	2	7
Mentor meeting **D** N=10				1	9
Next steps **E** n =18			1	1	20

Time allocation was appropriate

**Time allocation was appropriate**	**Strongly disagree**	**Disagree**	**Unsure**	**Agree**	**Strongly agree**
**Clinical areas**					
Training Plan				4	21
Pharmaceutical care planning			5	5	15
Efficient Prescribing and error management **A** n=16		1	2	3	9
Antipsychotic medicines in care homes			1	3	21
Covert administration and dysphagia			1	5	15
Care home Medicine **B** N=11			3	3	9
Complex patient case studies				6	18
PIP Case studies			2	7	16
**Research procedures**					
CHIPPS overview			1	4	20
Research issues & practical procedures				5	20
CHIPPS trial Internal Pilot and RCT				6	19
Capacity assessment and family involvement **C** n=10			1	1	8
Mentor meeting **D** N=10				1	9
Next steps **E** n =18			1	1	20

Key

A Only pilot, phase 1 and 2 N= 16 PIPs

B Only phase 2 and 3 P N=15 PIPS

C Only pilot and phase 1 N= 10 PIPS

D Only pilot and phase 1 n= 10 PIPS

E missing data from phase 2 n=3
